# Motion Preserving Surgery for Degenerative Cervical Myelopathy: Where Are We Now?

**DOI:** 10.1177/21925682251334092

**Published:** 2026-01-05

**Authors:** Husain Shakil, Shekar Kurpad, Jefferson R. Wilson

**Affiliations:** 1 Division of Neurosurgery, Department of Surgery, 7938 University of Toronto, Toronto, ON, Canada; 2 Division of Neurosurgery, St. Michael’s Hospital, Toronto, ON, Canada; 3 Institute of Health Policy Management and Evaluation, 7938 University of Toronto, Toronto, ON, Canada; 4 Department of Neurological Surgery, 5506 Medical College of Wisconsin, Milwaukee, WI, USA

Degenerative Cervical Myelopathy (DCM) is a ubiquitous and Heterogeneous disease which most spine surgeons encounter on a daily basis.^1^ DCM can arise secondary to a number of age-related pathologies afflicting the cervical spine, including spondylosis, osteophytosis, disc degeneration, and hypertrophy or ossification of soft tissues.^2^ These pathologies can injure the cervical cord through both static and dynamic mechanisms. Static factors include reduced cross-sectional area resulting in canal stenosis and chronic cord compression that is suspected to give rise to a cascade of local hypoperfusion, ischemia, and neuroinflammation.^3^ Conversely, dynamic factors, such as regional instability from degeneration, are thought to cause repeated trauma to the cervical cord during spinal motion, resulting in a chronic spinal cord injury phenotype.^4^

In light of increased disease recognition, early diagnosis related to improved access to spinal MRI, and the overall aging of the population, the incidence and prevalence of DCM is likely to increase further in coming years, creating a strong imperative to create improved treatment solutions.^4^ Presently, surgery is the only effective treatment for DCM consistently shown to improve patient quality of life and physical function.^5-7^ Historically, decompression and instrumented fusion, whether accomplished from an anterior, posterior or combined approach, has represented the mainstay of surgical treatment for DCM.^5,6,8^ Limiting motion has been previously thought to counteract dynamic mechanisms of injury to the cervical cord suspected to occur among DCM patients.^4,9^ However the negative biomechanical consequences of arthrodesis, in the way of adjacent segment degeneration is an all too frequent occurrence, often necessitating more complicated revision procedures.^10^ Moreover, loss of range of motion to the cervical spine has been found to impact activities of daily living, which can be expected to impair quality of life.^11^

Accordingly, there has been recent interest in techniques for motion preservation after surgery for degenerative cervical disease.^12^ Techniques for motion preservation include cervical disc arthroplasty (CDA), endoscopic cervical spine surgery, laminectomy without fusion, and laminoplasty. However, the evidence and considerations of these techniques within the context of DCM is rarely specified.

In this special joint focus issue from the AO Spine North America Research Committee and the AO Spine Spinal Cord Injury Knowledge Forum, we feature 6 papers that review and synthesize the evidence regarding motion preserving surgery for DCM. The first article presents a systematic review of 17 studies examining outcomes following DCM treatment with CDA. This review focuses on the incidence and predictors of persistent postoperative myelopathy, functional outcomes, pain scores, range of motion, alignment, and complications, while also comparing CDA with anterior cervical discectomy and fusion. The second paper systematically reviews 12 studies including patients treated with endoscopic cervical spine surgery (ECSS) for cervical stenosis, and reviews clinical outcomes, complications, and available techniques for ECSS. The third paper compares clinical outcomes between laminectomy alone and laminectomy with fusion and estimates the incidence of post-laminectomy kyphosis in patients with DCM through a systematic review and meta-analysis of 25 studies. The fourth paper systematically reviews and meta-analyzes evidence comparing laminoplasty with laminectomy and fusion for DCM treatment, focusing on healthcare costs, pain scores, postoperative opioid use, and return to work. The fifth paper presents a technical review of laminoplasty techniques, with considerations related to various DCM etiologies. Finally, the sixth paper provides a scoping review that compares outcomes following skip laminectomy to those after laminoplasty, in addition to reviewing outcomes and complications associated with oblique corpectomy in DCM patients. Collectively, these contributions clarify the evidence supporting motion preservation in the treatment of DCM and serve as a valuable reference for practitioners managing this prevalent condition.
